# Iron and breast cancer risk

**DOI:** 10.1186/1897-4287-10-S4-A5

**Published:** 2012-12-10

**Authors:** Grzegorz Sukiennicki, Magdalena Muszyńska, Tomasz Huzarski, Jacek Gronwald, Cezary Cybulski, Tadeusz Dębniak, Aleksandra Tołoczko-Grabarek, Oleg Oszurek, Anna Jakubowska, Antoni Morawski, Jan Lubiński

**Affiliations:** 1 Read-Gene SA and Pomeranian Medical University, Szczecin, Poland

## 

The aim of the study is identification of correlations between the serum concentrations of iron and the risk of breast cancer among female BRCA1 mutations carriers and unselected breast cancer patients.

The first group (99 triplet) selected for the trial were Polish women, positive for at least one of three founder mutations in BRCA1 gene dominating in Poland (5382insC, C61G, 4153delA). Serum was collected at the time of breast cancer diagnosis. Persons with detected tumor were considered as cases and the others were considered as controls. One case and two controls were paired regarding many criteria (e.g. age, family cancer history, cigarettes smoking, adnexectomy status ) to achieve the maximum of similarity between them.

The subjects of the second group (28 triplet) selected for the trial were Polish women, positive for at least one of three founder mutations in BRCA1 gene. Serum was collected 1-2 years before breast cancer diagnosis. One case and two controls were matched for year of birth, past history of cancer, adnexectomy status and cigarettes smoking to achieve the maximum of similarity between them.

The subjects of the third group were unselected cancer for the trial were Polish women. Serum was collected during breast cancer diagnosis before treatment. One case and one control were matched for year of birth (+/- 3 years), number and location of cancer among Iº relatives, smoking-the number of pack years (+/- 10%), adnexectomy status, CHEK2 mutation.

The proportion of cases and control in the first quartile was taken as a reference to calculate the odds ratio, confidence interval and p-value of the multivariate conditional logistic regression.

The iron was quantitatively measured by ICP-MS (Inductively Coupled Plasma Mass Spectrometry), (model Elan DRC-e 6100 th, PerkinElmer).

This study shows that concentration levels of iron in blood serum are a strong factors associated with an additionally increased risk of breast cancer among BRCA1 mutation carriers and unselected breast cancer patients.

For iron concentration in BRCA1 carriers (serum collected at the time of breast cancer diagnosis) all quartiles above the first one had a decreased risk of breast cancer. The results are shown in Table 1.

**Table 1:** Association between Fe serum concentration and risk of breast cancer in BRCA 1 carriers (serum collected at the time of breast cancer diagnosis).

Fe (µg/l)	Cases (n=99)	Controls (n=198)	OR	p-value
370,95 – 977,64	34 (34,3%)	40 (20,2%)	1,000	-
977,64 – 1262,78	24 (24,2 %)	50 (25,3%)	0,565	0.01229
1262,78 – 1571,11	24 (24,2%)	50 (25,3%)	0,565	0.13885
1571,11 - 4756,15	17 (17,17%)	58 (29,3%)	0,345	0.00823

For iron concentration in BRCA1 carriers (serum collected 1-2 years before breast cancer diagnosis) the last quartile had a decreased risk of breast cancer. The results are shown in Table 2.

**Table 2:** Association between Fe serum concentration and risk of breast cancer in BRCA 1 carriers (serum collected 1-2 years before breast cancer diagnosis).

Fe (µg/l)	Cases (n=27)	Controls (n=56)
313,90 – 945,79	9 (33,3%)	12 (21,4%)
954,79 – 1157,15	9 (33,3%)	12 (21,4%)
1158,32 – 1437,54	5 (18,5%)	16 (28,6%)
1460,00 – 2193,10	4 (14,8%)	16 (28,6%)

Individuals classified in the III and IV quartile had lower risk of breast cancer however results were statistically insignificant.

The strongest result and lowest cancer risk was for Fe > 1350 μg/l (OR= 0,289, p= 0,0426)

For iron concentration in unselected breast cancer patients (serum was collected during breast cancer diagnosis before treatment) all quartiles were statistically insignificant. The results are shown in Table 3.

**Table 3:** Association between Fe serum concentration and risk of breast cancer

Fe (µg/l)	Cases (n=151)	Controls (n=151)
86,47 – 813,58	43 (28,47%)	32 (21,19%)
813,92 – 992,11	40 (26,49%)	35 (23,18%)
993,06 – 1265,75	35 (23,18%)	41 (27,15%)
1268,27 – 3676,38	33 (21,85%)	43 (28,48%)

Individuals classified in the III quartile had lower risk of breast cancer however results were statistically insignificant.

The strongest result and lowest cancer risk was for Fe > 1000 μg/l (OR= 0,6698, p= 0,25554).

For iron concentration in unselected breast cancer patients (serum was collected during breast cancer diagnosis before treatment) before 55 years old, third quartile had a decreased risk of breast cancer. The results are shown in Table 4.

**Table 4:** Association between Fe serum concentration and risk of cancer in group of patients < 55 years old.

Fe (µg/l)	Cases (n=59)	Controls (n=62)
127,58 – 725,31	19 (32,2%)	11 (17,7%)
752,60 – 987,19	17 (28,8%)	13 (21,0%)
987,19 – 1285,18	9 (15,3%)	21 (33,9%)
1294,72 – 2584,76	14 (23,7%)	17 (27,4%)

Individuals classified in the III quartile had lower risk of breast cancer (OR=0,248, p=0,019);

The strongest result and lowest cancer risk was for Fe concentration higher than 987 μg/l (III quartile) ; max 1285 μg/l (III quartile)

